# A unique presentation of bilateral kissing molars and three‐rooted maxillary premolars: A case report and review of literature

**DOI:** 10.1002/ccr3.4679

**Published:** 2021-08-21

**Authors:** Krishan Sarna, Ian Murithi, Florence Opondo, Symon Guthua

**Affiliations:** ^1^ Division of Oral and Maxillofacial Surgery Department of Oral and Maxillofacial Surgery, Oral Pathology and Oral Medicine University of Nairobi Nairobi Kenya; ^2^ Division of Dental and Maxillofacial Radiology Department of Oral and Maxillofacial Surgery University of Nairobi Nairobi Kenya

**Keywords:** bilateral kissing molars, bilateral three‐rooted maxillary premolars, CBCT, impaction, rosetting molars

## Abstract

3D radiographic evaluation of the patient should be conducted before disimpaction to establish proximity to the mandibular canal and borders of mandible. In addition, variations of the root canal system of premolars must thoroughly be understood.

## Graphical abstract

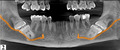

## 1 INTRODUCTION

A bilateral occurrence of kissing molars and three‐rooted maxillary second premolars is rare and not been reported previously in literature. In this report, we describe the detailed clinical and radiographic evaluation, including considerations of key factors prior to any surgical or endodontic intervention in such a patient.

Tooth impaction is a pathological process whereby the tooth does not erupt into its functional position due to physical or genetic factors.^1^ The most commonly affected teeth are the mandibular third molars having a prevalence of 16% to 68% while the least common are the mandibular second molars whose prevalence is 0% to 2.3%.^2^, ^3^, ^4^ The “kissing molar” (occlusal surfaces contacting each other with their roots pointed in opposite directions) relationship of these teeth is extraordinarily sparse with only eleven cases of bilateral pathology having been reported to date.^5^, ^6^ The etiopathogenesis of this condition remains unknown; however, arch length deficiency and metabolic diseases are implicated.^5^, ^7^, ^8^

Abnormal root morphology of the maxillary premolars, although uncommon, has been reported previously in literature involving relatively rare cases such as presence of a third root of the first maxillary premolar and a two‐rooted second maxillary premolar in about 25% of cases.^9^, ^10^ There are scarce reports on the occurrence of a three‐rooted second maxillary premolar whose incidence was reported to be 0.3% to 2% with no reports on a bilateral occurrence of this phenomenon.^11^, ^12^

We hereby report a unique combination of two rare dental findings in the same individual which could pose future treatment challenges: bilateral kissing molar impactions and bilateral three‐rooted second maxillary premolars. In addition, we discuss the relationship of the molar apices to the mandibular neurovascular bundle and to the inferior border of mandible, its surgical implications and the endodontic considerations of a three‐rooted second maxillary premolar.

## 2 CASE PRESENTATION

A 27‐year‐old African male patient presented to the Department of Oral and Maxillofacial Surgery, University of Nairobi, Kenya, with spontaneous, severe pain of left mandible for 7 days. Intraoral examination revealed bilateral Angle's class 2 molar relationship. The maxillary canines were missing (disimpactions performed 10 years ago), and carious lesions were present on 17 and 36 with both teeth tender to percussion.

**FIGURE 1:**
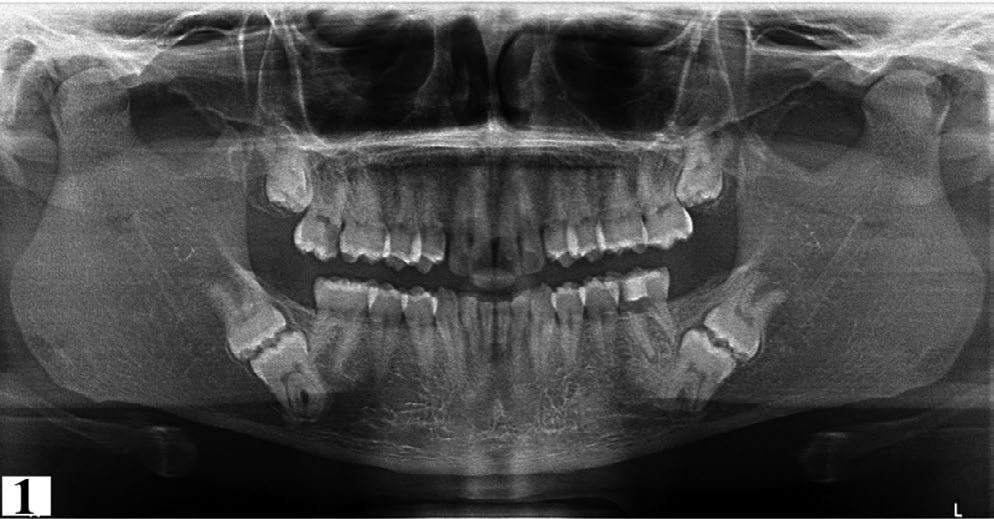
Panoramic radiograph illustrating bilateral maxillary molar impactions, missing maxillary canines and mandibular KMs. Radiolucencies can be seen on 17 and 36 mesially

**FIGURE 2:**
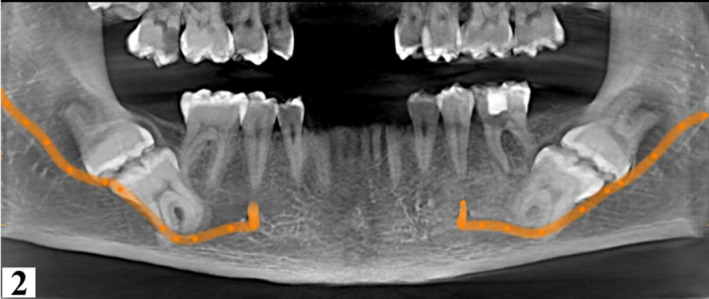
Reformatted panoramic at 14 mm slice thickness showing the course of the mandibular canals which transmit the mandibular neurovascular bundle. The second mandibular molar roots were bilaterally contiguous with the canal

**FIGURE 3:**
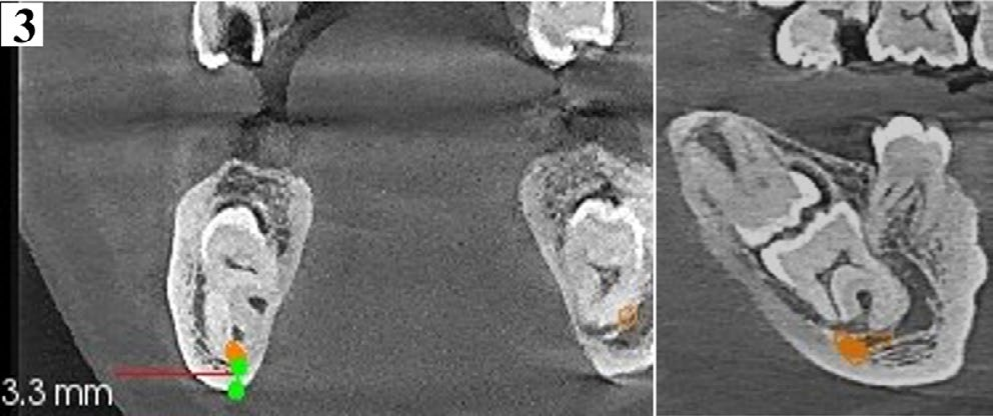
Orthogonal coronal slice (left) and corresponding sagittal slice (right) at 150 microns confirm contact between the inferior alveolar nerve (orange dot) and roots of 47. Thin lingual cortex is noted as well as close proximity to inferior border of mandible (3.3 mm)

**FIGURE 4:**
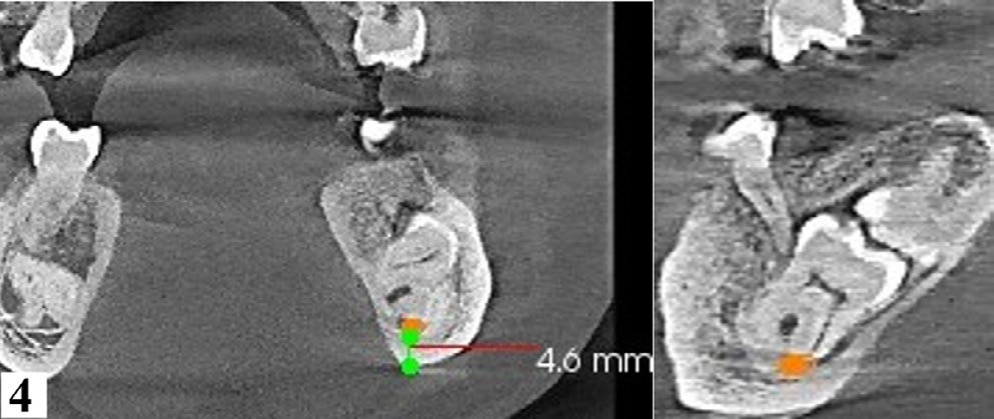
Orthogonal coronal slice (left) and corresponding sagittal slice (right) at 150 microns confirm contact between the inferior alveolar nerve (orange dot) and roots of 37. Thin lingual cortex is noted as well as close proximity to inferior border of mandible (4.6 mm)

**FIGURE 5:**
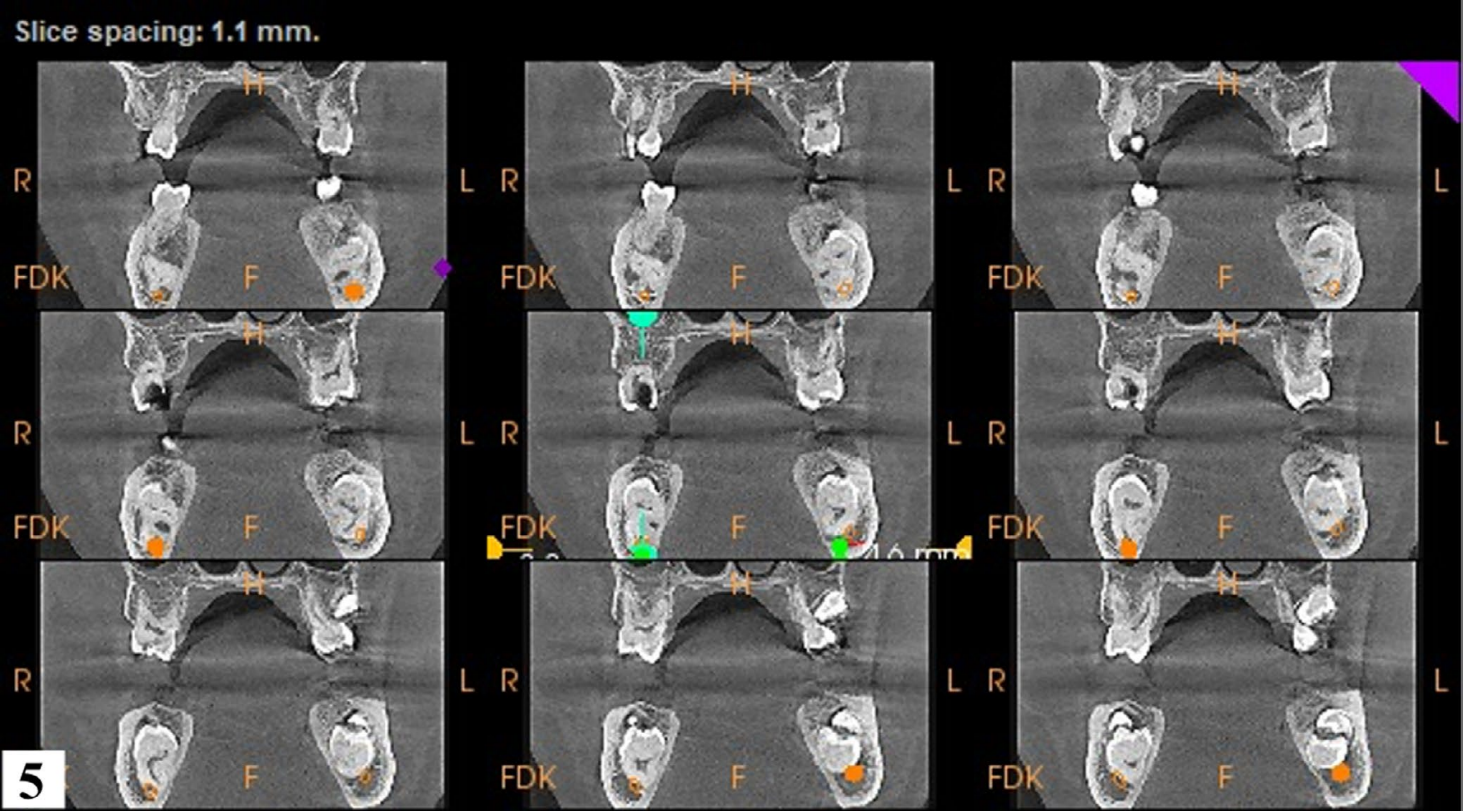
An illustration of the split view orthogonal coronal sections employed in assessing the path of the inferior alveolar nerve

**FIGURE 6:**
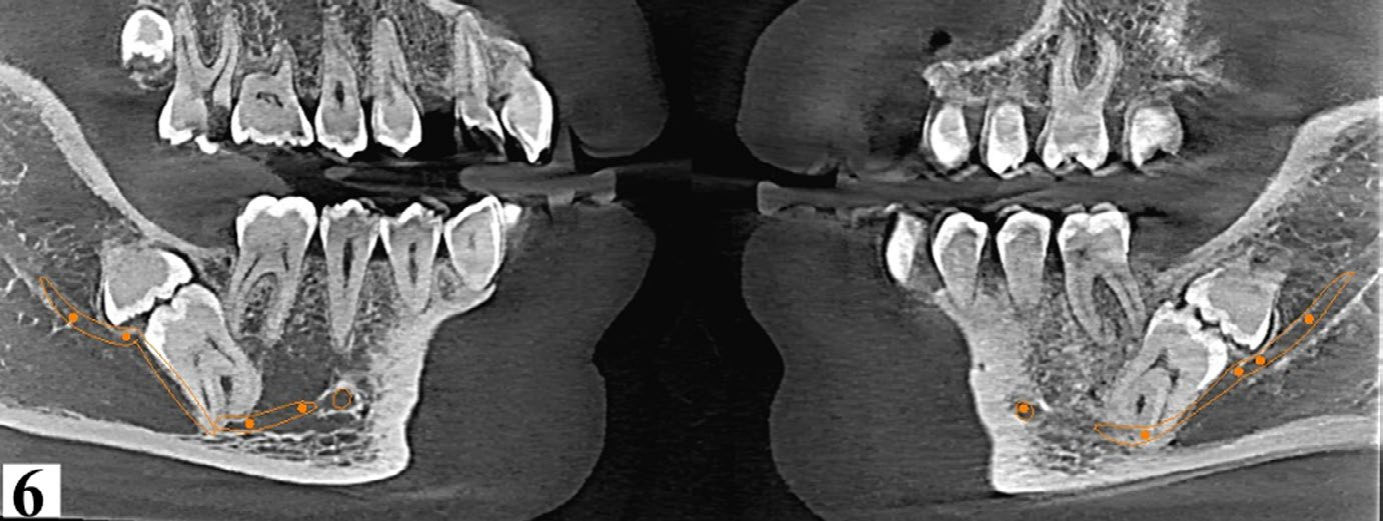
Sagittal oblique sections at 150 microns showing the nerve track and relationship with molar apices on both right (left image) and left (right image) sides of the patient

**FIGURE 7:**
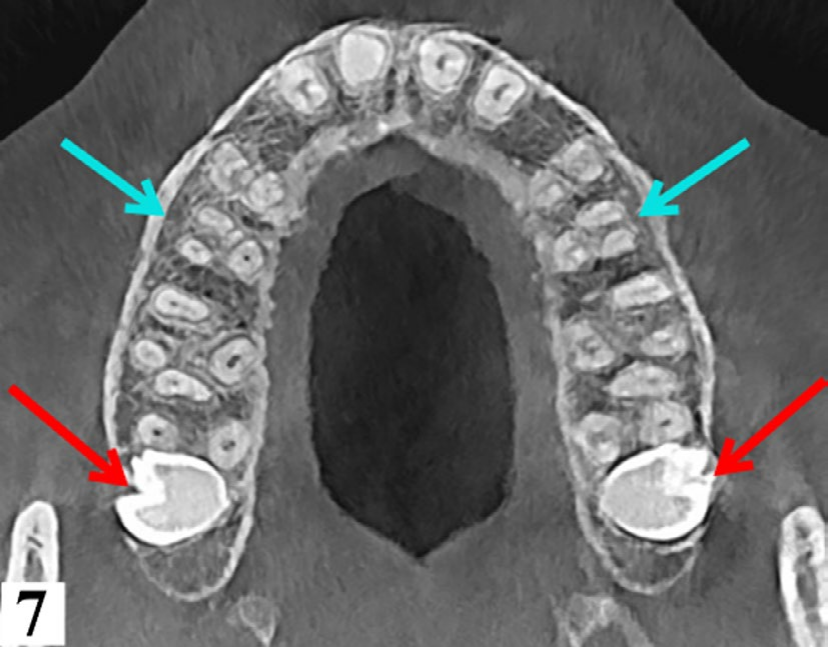
Axial view illustrating three‐rooted maxillary second premolars (blue arrows) and transverse orientation of impacted 18 and 28 (red arrows)

A panoramic radiograph revealed radiolucencies with probable pulpal involvement on the mesial aspect of 17 and 36. The maxillary third molars were impacted. Mandibular second and third molars (37, 38, 47, 48) were impacted bilaterally with their occlusal surfaces in contact and roots pointed in opposite directions (Figure 1). In order to evaluate the nature of the molar impaction, proximity to mandibular canal and bucco‐lingual positioning within the mandible, a cone beam computed tomography (CBCT) scan was performed (Figures 2, 3, 4, 5, 6, 7). The imaging parameters were as follows: CS 8200 3D (12X10), Voltage, 90kV; exposure time, 10 s; current 5.0 mA; voxel size 150 microns.

A reconstructed panoramic was generated using CS Version 8 software. Tracking of the mandibular canal was done by applying the color tracer to the smallest slice thickness of 15 microns to illustrate the entire path of the canal in one view (Figure 2). Coronal sections confirmed contact between the mandibular canal and roots of 37 and 47. For reproducibility of measurements, only reference (main) orthogonal planes were examined in split views of 5x5, while oblique sections were only employed for visual illustration. Additionally, the distal roots of the 37 and 47 were 4.6 mm and 3.3 mm, respectively, from the inferior border of the mandible. (Figures 3 and 4). Thinning of the lingual cortex and paucity of cancellous bone lingual to the 37 and 47 were observed. Cortical perforation was, however, ruled out. Curved roots of second mandibular molars were noted which were in contact with the mandibular canal. (Figures 3, 4, 5, 6).

Axial sections of the maxilla imaging revealed three‐rooted second maxillary premolars (15 and 25). Both had divergent mesiobuccal, distobuccal, and palatal roots with corresponding root canals. (Figure 7) The patient was managed conservatively by endodontic treatment of the carious teeth (17 and 36). Due to the asymptomatic nature of presentation, he was advised on disimpaction of impacted maxillary molars while long‐term follow‐up was recommended for the KMs.

## 3 DISCUSSION

KMs or “rosette formation” is a rare phenomenon first described in 1973 where the molars are in occlusion within a single, enlarged follicular space.^13^ The incidence of KMs has been reported to be 0.06%, being higher in males with an age range of 13–58 years.^5^, ^8^ Majority are unilateral and occur in the mandible, although bilateral cases have been reported.^6^, ^13^, ^14^, ^15^, ^16^, ^17^, ^18^, ^19^ In the present case, the patient presented with bilateral KMs as an incidental finding. Past medical history may be significant in the occurrence of KMs which may imply that they are a manifestation of diseases rather than independent entities occurring in otherwise healthy individuals. Notably, mucopolysaccharidosis is implicated in some cases with KMs.^18^ Existing literature was reviewed extensively and a summary of the type of KMs, symptoms, associated diseases, and treatment presented (Table 1).

**TABLE 1:** Summary of present literature on KMs

Author	Age	Sex	Symptoms	Molar Impaction	Associated diagnosis/ pathology	Treatment	Post Operative Complications
Anish N et al ^17^	35	M	None	37,38; 47,48	None	Maintained	–
Bakaeen G, Baqain Zh et al^14^	23	M	Facial Pain	37,38; 47,48	None	Surgical Removal Under GA	Trismus And Dry Socket
Gulses A et al ^15^	32	F	None	7 and 8 unilateral (NS)	Dentigerous Cyst	Surgical Removal	Uneventful
Gulses A et al ^15^	23	M	None	7 and 8 unilateral (NS)	Granulomatous Changes Of Follicle	Surgical Removal	Paresthesia Of IAN
Gulses A et al ^15^	22	F	None	7 and 8 unilateral (NS)	Granulomatous Changes Of Follicle	Surgical Removal	Paresthesia Of IAN
Gulses A et al ^15^	20	F	None	7 and 8 unilateral (NS)	None	Extraction	Uneventful
Kiran HY et al ^5^	18	F	None	37,38; 47,48	None	Surgical Removal Under GA	Uneventful
Krishnan B et al ^25^	36	F	Swelling	37,38	Dentigerous Cyst	Extraction Under LA	Uneventful
Manani A et al ^24^	38	M	None	37,38	None	Maintained	–
Mcintyre G et al ^23^	19	F	Pericoronitis	37,38	None	Extraction Under LA	Trismus, Dry Socket
Nakamura et al ^18^	25	M	None	37,38; 47,48	Mucopolysaccharaidosis	Maintained	–
Nakamura et al ^18^	17	M	None	37,38; 47,48	Mucopolysaccharaidosis	Maintained	–
Nakamura et al ^18^	21	M	None	37,38; 47,48	Mucopolysaccharaidosis	Maintained	–
Gonzalez‐Perez et al^26^	29	F	Swelling	47,48	None	Surgical Removal Under GA	Uneventful
Gonzalez‐Perez et al^26^	35	M	Pain In TMJ	47,48	Dentigerous Cyst	Surgical removal under GA	Uneventful
Robinson Ja et al ^6^	25	M	None	37,38; 47,48	No Relevant History	Maintained	–
Sa Fortes et al ^16^	33	M	None	37,38; 47,48	Dentigerous Cyst	Surgical Removal Under GA	Uneventful
Van Hoof (1973)^13^	31	M	None	37,38; 47,48	Mental Retardation	Maintained	–
Zerener et al ^19^	–	–	Swelling	37,38; 47,48	None	Surgical Removal under GA	Uneventful
**Present Case**	**27**	**M**	**None**	**37,38; 47,48**	**None**	**Maintained**	**–**

Abbreviations: GA, general anesthesia; LA, local anesthesia; NS, not specified; TMJ, temporomandibular joint.

Management of symptomatic KMs necessitates surgical intervention while that of asymptomatic KMs entails maintenance of the molars within the jaw.^20^ Maintenance could lead to complications such as reduction in mandibular bone mass over time hence increasing risk of fracture, dentigerous cyst formation, root resorption, pericoronitis, compression of the inferior alveolar nerve (IAN) leading to paresthesia of the lip and functional impairment.^7^, ^15^, ^21^, ^22^ On the contrary, complications may occur intra‐operatively and post‐operatively following surgical disimpaction. These include fracture of the mandible, damage to IAN and lingual nerve, temporomandibular joint disorders, and infections.^14^, ^15^, ^19^, ^23^, ^24^, ^25^, ^26^ As such, the patient should be informed of the complications and consent obtained prior to surgery. Such cases, therefore, necessitate referral and review by a specialist oral and maxillofacial surgeon with comprehensive clinical and radiographic investigations done prior to management.^19^ As pertains the current case, there was contact between 37 and 47 root apices with the IAN and curvature of the tooth roots. Additionally, the distance to the inferior border of mandible was considerably reduced hence increasing the risk of fracture during surgical disimpaction. These findings informed the decision to maintain the KMs within the jaw and review the patient annually.

Anatomic variations of maxillary second premolars are well documented in the literature; however, trifurcated second premolars are rare.^27^, ^28^, ^29^, ^30^, ^31^ (Table 2) Knowledge of such variation is crucial during endodontic treatment of the premolar which may necessitate modification of the typical access cavity and technique of management.^28^, ^32^ Visualization of the third root (palatal root) of the premolar can be challenging on plain radiographs due to superimposition of the buccal roots.^33^, ^34^ The panoramic image (Figure 1) is a good example of the latter, whereby superimposition of the mesiobuccal roots concealed the palatal roots. If the mesiodistal width of the mid‐root image is equal to or greater than the mesiodistal width of the crown, three canals should be suspected.^35^ The clinician should have a thorough understanding of the pulp chamber anatomy and root canal system along with possible departures from the norm prior to performing any endodontic therapy. Moreover, good illumination and magnification can greatly improve canal visualization and management of complex root canal systems.^36^

**TABLE 2:** Summary of present literature reports on three‐rooted second premolars

Author	Age	Sex	Ethnicity	Laterality	Tooth
Gomez et al., 2009^27^	32	M	Brazilian	Unilateral	25
Sathyanarayan et al.,2017^28^	24	M	Indian	Unilateral	15
Kumar et al., 2015^29^	27	M	India	Unilateral	15
Alwehaiby et al., 2020^30^	22	M	Saudi Arabia	Unilateral	15
Shetty et al., 2014^31^	37	F	India	Unilateral	15
**Present case**	**27**	**M**	**Kenya**	**Bilateral**	**15, 25**

## 4 CONCLUSION

A thorough clinical and radiographic evaluation is a key in ensuring adequate management. Whereas panoramic imaging is considered the gold standard, CBCT is highly recommended in order to evaluate the 3D position of such impactions and their relationship with critical structures before surgical intervention. Taking all diagnostic factors of this case into account, long‐term follow‐up was recommended due to the proximity of the molar apices to the IAN and inferior border of mandible.

## CONFLICT OF INTEREST

No conflict of interest to declare.

## AUTHOR CONTRIBUTIONS

Krishan Sarna and Ian Murithi collected the data and wrote the findings of the manuscript while Dr. Florence Opondo and Professor Symon Guthua supervised and provided critical guidance in radiologic assessment, interpretation and final preparation of the manuscript. All authors approved the final manuscript.

## ETHICAL APPROVAL

The manuscript was prepared according to standard publication ethical guidelines.

## Data Availability

The data that support the findings of this study are available on request from the corresponding author. The data are not publicly available due to privacy or ethical restrictions.
